# Fabrication and Characterization of Flexible Capacitive Humidity Sensors Based on Graphene Oxide on Porous PTFE Substrates

**DOI:** 10.3390/s21155118

**Published:** 2021-07-28

**Authors:** Zhenyu Wei, Jianqiu Huang, Wenhao Chen, Qingan Huang

**Affiliations:** Key Laboratory of MEMS of the Ministry of Education, Southeast University, Nanjing 210096, China; 230198813@seu.edu.cn (Z.W.); chenwhcn@163.com (W.C.)

**Keywords:** humidity sensor, flexibility substrate, polytetrafluoroethylene (PTFE), hydrophobic

## Abstract

Porous polytetrafluoroethylene (PTFE) is physically flexible, thermally and chemically stable, relatively inexpensive, and commercially available. It is attractive for various flexible sensors. This paper has studied flexible capacitive humidity sensors fabricated on porous PTFE substrates. Graphene oxide (GO) was used as a sensing material, both hydrophobic and hydrophilic porous PTFE as the substrates, and interdigitated electrodes on the PTFE substrates were screen-printed. SEM and Raman spectrum were utilized to characterize GO and PTFE. An ethanol soak process is developed to increase the yield of the humidity sensors based on hydrophobic porous PTFE substrates. Static and dynamic properties of these sensors are tested and analyzed. It demonstrates that the flexible capacitive humidity sensors fabricated on the ethanol-treated hydrophobic PTFE exhibit high sensitivity, small hysteresis, and fast response/recovery time.

## 1. Introduction

Flexible humidity sensors have recently gained attention in various applications including respiratory devices, incubators, sterilization processes, medicine production, and storage [1,2]. The sensors with both cost-effective fabrication processes and high performance such as high sensitivity and linearity, low hysteresis, and fast response time are essential to meet the requirements of the emerging application areas. To address this need, the humidity sensors based on flexible substrates such as polyimide (PI), polydimethylsiloxane (PDMS), polyester (PES), polyethylene naphtholate (PEN), polyethylene terephthalate (PET), cellulose, and paper have been developed [2].

Flexible materials are mainly used as sensing layers and substrates, which meets robust, inexpensive, highly sensitive, and rapid response. Porous polytetrafluoroethylene (PTFE) is a polymer-based membrane that is breathable and physically flexible. It has not only thermal stability, chemical stability, and tear resistance, but also relatively low cost and availability in the commercial market. The porous PTFE has a large surface area, which makes it attractive for various flexible gas and humidity sensors. The hydrophobic porous PTFE has recently been utilized as the flexible substrate for gas sensors [3,4,5], in which hydrophobic and porous properties of the PTFE greatly enhance gas diffusion, leading to high sensitivity and fast response. As the substrate of humidity sensors, the hydrophobicity of PTFE surface improves the performance of the sensor, whereas it also causes the poor adhesiveness with metal and difficulty of fabrication. The hydrophilic porous PTFE has also been utilized recently for the flexible humidity sensors, in which the porous PTFE was used as both flexible substrates and sensing layers via resistive [6] or capacitive measurements [7]. However, the humidity sensors based on the hydrophilic PTFE substrates have large hysteresis and long response/recovery time.

Graphene oxide (GO) as the derivative of two-dimensional material graphene, contains a large number of oxygen-containing groups [8], which enhances the hydrophilicity and sensitivity to water molecules. Various humidity sensors using GO as the sensitive material with different structures and sensing principles have been proposed, such as the GO-Si bi-layer stress-based sensor [9], the CMOS interdigital capacitive humidity sensors [10], QCM humidity sensors [11], SAW humidity sensors [12], FBAR humidity sensors [13], humidity sensors based on GO foam [14], etc. The humidity sensors with the sensitive material of GO exhibit high sensitivity, wide range, and fast response/recovery time [15,16]. Our group attempted to use humidity sensing layers such as PI and GO fabricated on porous PTFE substrates for the capacitive flexible humidity sensors [17,18]. Due to poor adhesion of the hydrophobic surface [19], it is difficult to deposit materials on the PTFE substrate, which leads to a low yield of the sensors. Though the hydrophilic PTFE solves the problem of poor surface adhesion of the substrate, it causes the disadvantage of large hysteresis and long response time of humidity sensors.

In this paper, a capacitive flexible humidity sensor is presented in which GO is used as the sensing layers and a pair of Ag interdigitated electrodes are fabricated using a screen-printing process. In particular, the hydrophobic and hydrophilic porous PTFE as the flexible substrates are studied and compared. An ethanol treatment process is developed here to fabricate the humidity sensors on the porous hydrophobic PTFE substrates. The yield of sensors fabricated on the hydrophobic PTFE substrates is improved by this additional process without affecting the sensors performances. Compared with the traditional hydrophobic and hydrophilic PTFE substrates, the ethanol-treated porous hydrophobic PTFE substrate increases both yield and performances of the flexible capacitive humidity sensors.

## 2. Fabrication of the Humidity Sensors

**Figure 1:**
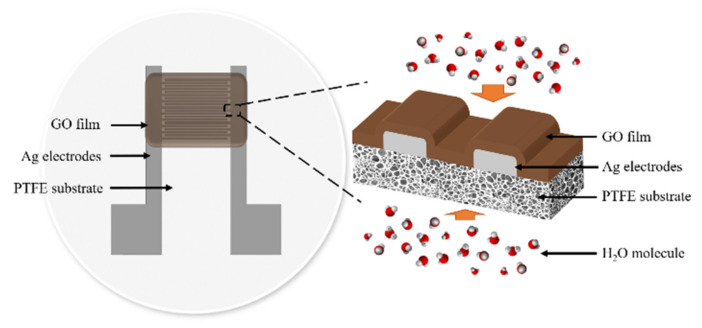
The structure of a flexible capacitive humidity sensor.

A flexible capacitive humidity sensor consists of a porous PTFE substrate, a pair of Ag interdigitated electrodes, and a GO sensitive film, as shown in Figure 1. The length of each electrode is 7 mm, the overlap length is 5 mm, and the electrode width and spacing are both 200 µm.

The fabrication processes of the flexible capacitive humidity sensors are as follows. (1) Silver layers were deposited and patterned as the interdigitated electrodes on the porous PTFE substrate by screen-printing process, then the sample was heated in vacuum at 80 °C for 30 min; (2) an aqueous dispersion of GO (0.04 mL, 1 mg/mL, XFNANO Technology, Nanjing, China) was dripped on the interdigitated electrodes, and then heated in vacuum at 50 °C for 1 h.

Both hydrophilic and hydrophobic PTFE (Xinya Purification Material Factory, Shanghai, China) are used as the substrates, respectively. PTFE is a kind of polymer material based on the skeleton of the polyethylene (PE) molecular chain, and the hydrogen atoms connected with the carbon atoms are all replaced by fluorine atoms. As each carbon atom is connected with two fluorine atoms, the highly symmetrical structure causes the low surface energy of PTFE, which results in the difficulty of poor printing of the surface of PTFE. When the surface of hydrophobic PTFE is treated by plasma or other methods, part of the carbon-fluorine bond is destroyed, and the active group replaces the fluorine atom. The surface of PTFE becomes hydrophilic, which enhances the surface adhesion with metal.

The silver layers are adhered much more easily on the hydrophilic surface than on the hydrophobic surface, which has a significant impact on the quality of screen-printing [19]. Due to the good wettability of ethanol on PTFE, a special ethanol treating progress was developed here for the hydrophobic PTFE substrate as follows. The hydrophobic PTFE substrate was immersed in ethanol at 25 °C for 3 h, and then dried at room temperature for 10 min before the screen-printing step. The ethanol being left over the surface can increase the adhesion of the substrate, which improves the yield and quality of screen-printing. Additionally, the ethanol is then volatilized by the heating treatment in vacuum after the screen-printing. The substrates of the humidity sensors recover hydrophobicity, which ensure the low hysteresis and fast response.

**Figure 2:**
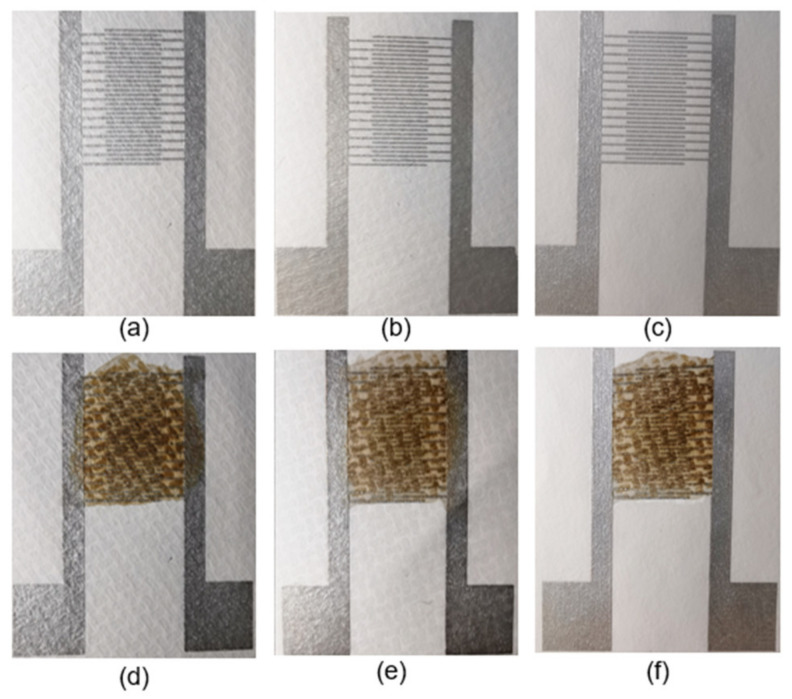
Photographs of the interdigitated electrodes on different substrates: (**a**) bare hydrophobic PTFE, (**b**) ethanol-treated hydrophobic PTFE, (**c**) hydrophilic PTFE. GO on the interdigitated electrodes: (**d**) bare hydrophobic PTFE, (**e**) ethanol-treated hydrophobic PTFE, and (**f**) hydrophilic PTFE.

The images of interdigitated electrodes and fabricated sensors on bare hydrophobic PTFE, ethanol-treated hydrophobic PTFE, and hydrophilic PTFE are shown in Figure 2, respectively. 

## 3. Experiments and Results

### 3.1. SEM

**Figure 3:**
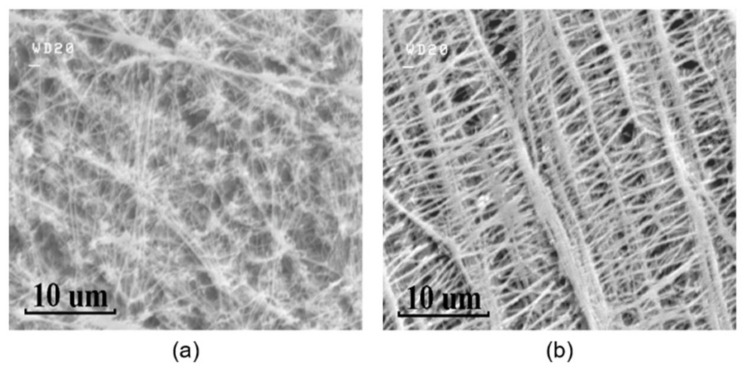
SEM images of the porous PTFE substrates: (**a**) hydrophobic PTFE, (**b**) hydrophilic PTFE.

The surface microstructures of the PTFE membranes and the GO films coated on different PTFE substrates are investigated with a scanning electron microscope (S-4500, HITACHI, Tokyo, Japan) at an accelerating voltage of 15.0 kV after the samples are coated with platinum. The SEM image of the porous hydrophobic and hydrophilic PTFE substrates are shown in Figure 3, respectively. Both hydrophobic and hydrophilic PTFE substrates have a random porous network structure, and as a result, water molecules can easily go through the network structures. Therefore, the back side of the substrate is also open to the humidity environment, as shown in Figure 1 so that the dynamic characteristics of the flexible capacitive humidity sensors can be improved.

**Figure 4:**
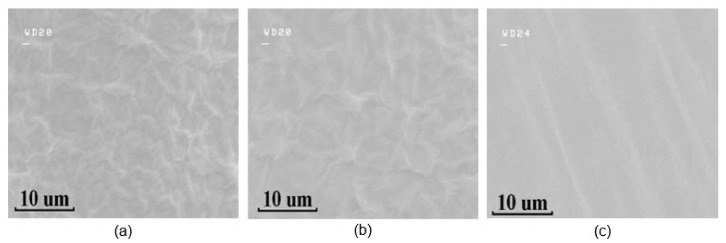
SEM images of GO on different substrates: (**a**) bare hydrophobic PTFE, (**b**) ethanol-treated hydrophobic PTFE, (**c**) hydrophilic PTFE.

The morphology of GO films on bare hydrophobic PTFE, ethanol-treated hydrophobic PTFE, and hydrophilic PTFE substrates are shown in Figure 4, respectively. The topographies of the GO films on bare hydrophobic PTFE and ethanol-treated hydrophobic PTFE are basically similar, whereas those on the hydrophilic surface are much more uniform. The folds of the GO films increase the contact area with water molecules, improving the sensitivity and response speed of the sensors.

### 3.2. Raman Spectrum

Compositions of the different PTFE and GO films on the different substrates are characterized by laser Raman spectrometer (HR800, HORIBA Jobin Yvon, Paris, France) with the 633 nm excitation wavelength of a 45 mW laser.

**Figure 5:**
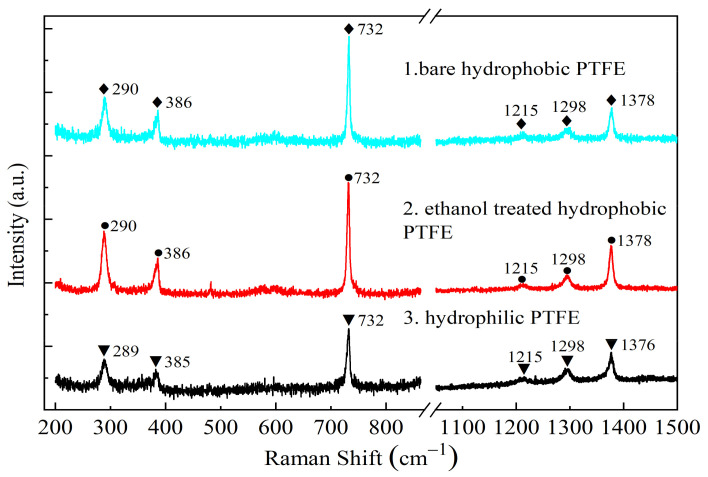
Raman spectrums of three kinds of PTFE: **1**. bare hydrophilic PTFE, **2**. ethanol-treated hydrophobic PTFE, **3**. hydrophobic PTFE.

The Raman spectrums of the surface of bare hydrophilic PTFE, ethanol-treated hydrophobic PTFE, and hydrophobic PTFE are shown in Figure 5. It can be seen that the bare hydrophobic PTFE and ethanol-treated hydrophobic PTFE have the same band positions and similar peak intensities at 290 cm^−1^, 386 cm^−1^, 732 cm^−1^, 1215 cm^−1^, 1298 cm^−1^, and 1378 cm^−1^. It means that the molecular structure of two kinds of PTFE is consistent, in other words, the material composites of the hydrophobic PTFE are not changed by the ethanol treating process.

The intensity of Raman active bands of the hydrophilic PTFE decreases obviously, especially the band at 732 cm^−1^. According to [20,21], the characteristic Raman active bands at 732 cm^−1^ assigned to stretching vibration of C-F, at 1378 cm^−1^ assigned to stretching vibration of C-C, while two bands at 290 cm^−1^ and 386 cm^−1^ attributed to deformation vibration of C-C in polymer chains, respectively. The significant reduction of the Raman peak intensity at 732 cm^−1^ means the ratio of fluorine groups of the hydrophilic PTFE is less than that of the hydrophobic PTFE, which confirms the principle that hydrophilicity of PTFE surface originates from the substitution of the active group to the fluorine group on the carbon chain.

**Figure 6:**
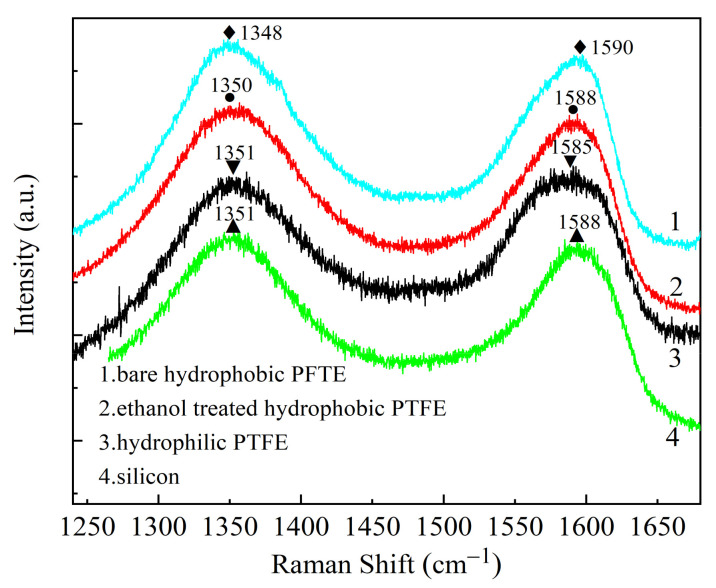
Raman spectrums of GO on different substrates: **1**. bare hydrophobic PTFE; **2**. ethanol-treated hydrophobic PTFE; **3**. hydrophilic PTFE; **4**. silicon.

The Raman spectrums of GO on different substrates are shown in Figure 6. For the GO film on a silicon (Si) substrate, the D peak is at 1351 cm^−1^, which is caused by the symmetrical stretching vibration of sp^2^ hybrid carbon atoms in the aromatic ring. The G peak is at 1588 cm^−1^, which is generated by the stretching motion of the carbon cyclic ring and all the sp^2^ atom pairs of the long chain [22]. Since the D peak is related to the defect activation, the intensity ratio of the D peak to the G peak (I_D_/I_G_) is a critical parameter to characterize the defect density of GO. The smaller the I_D_/I_G_, the higher the degree of atomic ordering and the fewer defects of GO. The software Origin 2018 was used to perform fitting using Gaussian function, and the peak ratio I_D_/I_G_ is calculated. The I_D_/I_G_ of GO on the substrates of bare hydrophobic PTFE, ethanol-treated hydrophobic PTFE, hydrophilic PTFE, and silicon are 1.067, 1.063, 0.990, and 1.064, respectively. It means compare with the hydrophobic substrate, the GO on the hydrophilic PTFE substrate has the fewest defects [22,23]. 

### 3.3. Static Water Contact Angles

**Figure 7:**
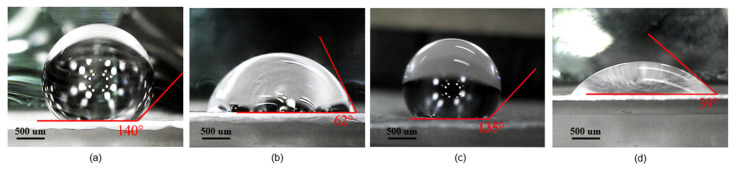
Water contact angle: (**a**) bare hydrophobic PTFE, (**b**) ethanol-treated hydrophobic PTFE (before heat treatment), (**c**) ethanol-treated hydrophobic PTFE (after heat treatment), (**d**) hydrophilic PTFE.

To compare the surface energy, the static water contact angles of different PTFE substrates were measured, as shown in Figure 7.

The contact angle of the bare hydrophobic PTFE is 140°, which means great hydrophobicity and lower surface energy of the surface, whereas the contact angle of the ethanol-treated hydrophobic PTFE (before heat treatment) and hydrophilic PTFE are 62° and 39°, respectively, which means good hydrophilicity and higher surface energy of these two substrates. After heat treatment, the surface of the ethanol-treated hydrophobic PTFE recovers the hydrophobicity, and the contact angle increases to 135°.

Generally, the surface with low surface energy is difficult to bond, whereas it is easy to deposit silver paste on a hydrophilicity surface. In other words, the hydrophilicity of the surface leads to higher yield of the screen-printing process. As mentioned in Section 2, ethanol remains on the surface of hydrophobic PTFE after ethanol treatment process, and the excellent hydrophilicity of ethanol makes the substrate surface temporarily hydrophilic, as shown in Figure 7b, which helps to improve the adhesion between the substrates and the silver paste during the screen-printing process. As a result, the ethanol does not permanently change the hydrophobicity of the PTFE surface. During the heating treatment after screen-printing, the ethanol completely evaporates, and the structure of the substrate remains unchanged, which is confirmed by the Raman test: the Raman spectral of the bare hydrophobic PTFE is as the same as that of the ethanol-treated hydrophobic PTFE. 

### 3.4. Yield of a Screen-Printing Process

**Figure 8:**
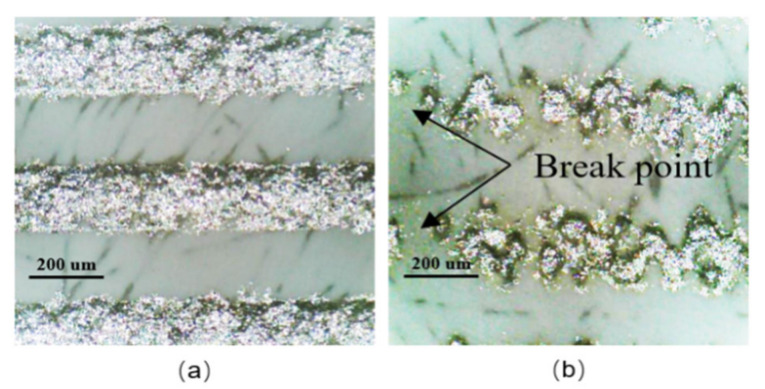
Typical morphology of interdigitated electrodes: (**a**) well-printed electrodes, (**b**) bad-printed electrodes.

The typical morphology of well-printed electrodes and bad-printed electrodes are shown in Figure 8, respectively. An intact electrode without breaking is considered as a well-printed electrode, as shown in Figure 8a. The well-printed silver electrodes are conductive, which can operate normally as interdigital capacitors. As shown in Figure 8b, there are break points in the middle of the silver electrodes, which cannot conduct normally, and the electrodes like these are considered as bad-printed electrodes.

The yield of the screen-printing process is defined as the ratio of well-printed electrodes to total ones, and the yield on different substrates can be obtained by observing the morphology of the electrodes under a microscope. The metal layers are adhered much more easily on the hydrophilic surface than on the hydrophobic surface [19]. According to our test, the yield of the screen-printing process on the ethanol-treated hydrophobic PTFE and hydrophilic PTFE substrates is relatively high, which is 75.33% and 85%, respectively. Whereas on the bare hydrophobic PTFE substrates, the yield is only 50.30%, which is lower than that on the ethanol-treated hydrophobic PTFE substrate. As mentioned in Section 3.3, ethanol being left over the PTFE makes the hydrophobic surface behave as hydrophilic during the screen-printing, which significantly improves the adhesion of the silver on the substrate and hence the yield. 

### 3.5. The Humidity Sensor Performance Test

The performance test of humidity sensors includes the static test and the dynamic test. In the test of characteristics, the output capacitance values are measured by a precision LCR meter (TH 2826A, Tonghui, Changzhou, China). The test frequency is 1 kHz. 

The static characteristics of fabricated GO humidity sensors was tested in a humidity chamber (C340, -70, Weiss-votesch, Giessen, Germany) at 25 °C in different humidity levels from 20% RH to 90% RH. The effect of temperature was also tested in the environmental chamber.

**Figure 9:**
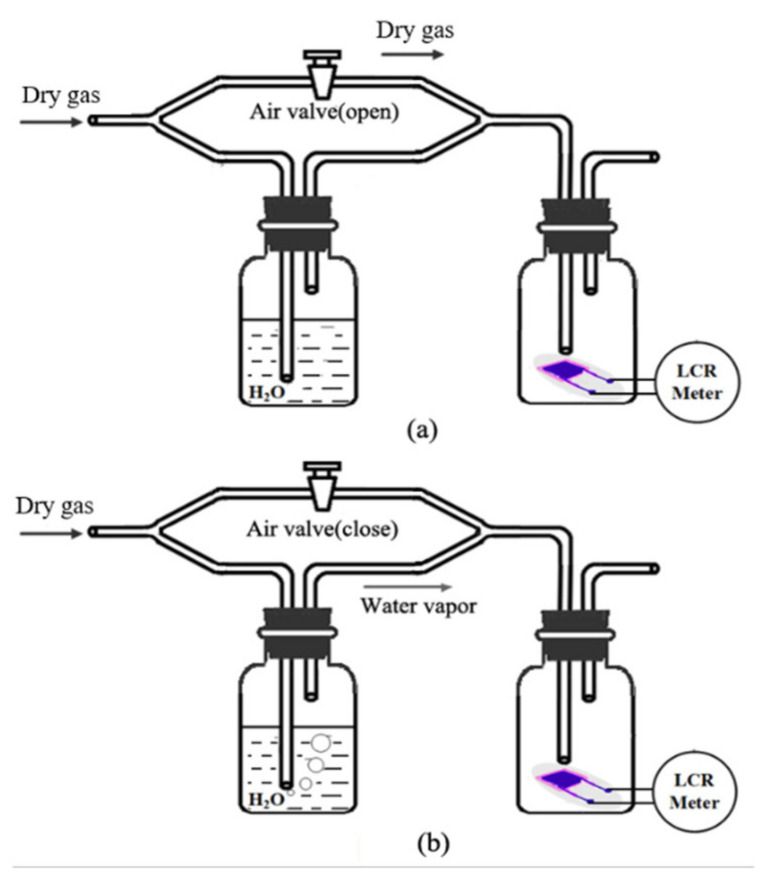
The dynamic test setup: (**a**) get dry gas (about 3% RH) when the valve is open; (**b**) get wet gas (about 96% RH) when the valve is closed.

The dynamic characteristics of the sensors are tested by the setup shown in Figure 9. An air valve is used to control flow paths and create a step change of relative humidity. As shown in Figure 9a, when the valve is open, the airflow goes through the pipes directly, so dry gas (about 3% RH, measured by M170 Hygrometer, Vaisala, Helsinki, Finland) is transferred onto the humidity sensor surface. When the valve is closed, the airflow passes through the deionized water. After mixed with water, the wet gas (about 96% RH) is conducted to the humidity sensor. The humidity changes between 3% RH and 96% RH rapidly with aid of the air valve. The dry gases are N_2_ and O_2_ (purity 99.999%, Shang-Yuan industrial gas plant, Nanjing, China). 

### 3.6. The Result of Static Characteristics

**Figure 10:**
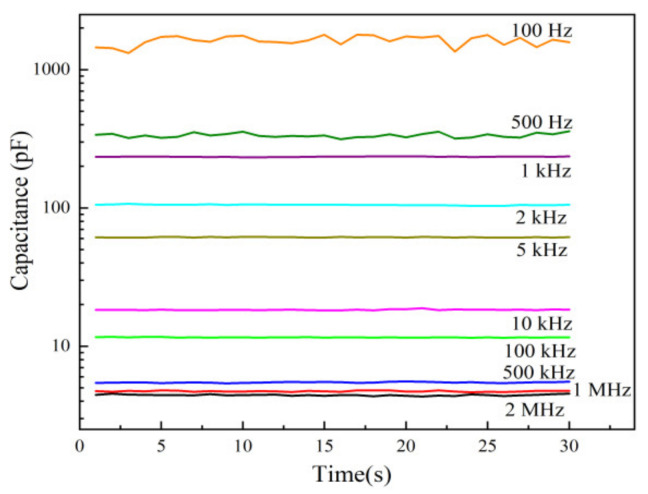
Capacitance stability of the sensors on ethanol-treated hydrophobic PTFE.

The capacitance of humidity sensors depends on the test frequency. As shown in Figure 10, the humidity sensor fabricated on an ethanol-treated hydrophobic PTFE substrate was tested at a series of frequencies from 100 Hz to 2 MHz under 25 °C and 60% RH. It can be observed that as the test frequency decreases, the capacitance of the sensor increases, while the noise is more significant at low frequencies. In order to get both large capacitance and low noise, 1 kHz is selected here as a test frequency. 

**Figure 11:**
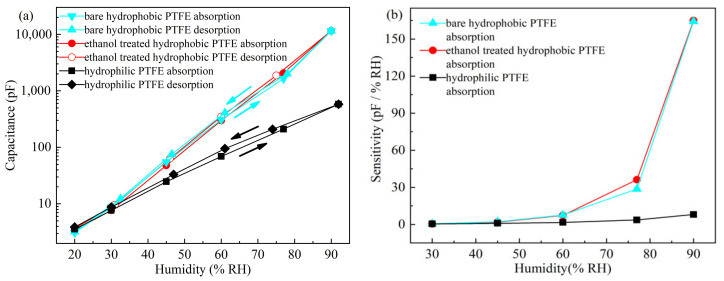
Static test results of the sensors on different substrates: (**a**) hysteresis; (**b**) sensitivity.

The hysteresis and sensitivity of the sensors on different substrates are shown in Figure 11a,b, respectively. The hysteresis properties of sensors on the bare hydrophobic PTFE and ethanol-treated hydrophobic PTFE are basically identical, and the maximum hysteresis is nearly 1% RH at 60% RH. The hydrophilic PTFE substrate, however, leads to relatively high hysteresis and low sensitivity. The maximum hysteresis is estimated to be around 4% RH at 60% RH. 

At low RH levels (<45% RH), sensors based on bare hydrophobic PTFE, ethanol-treated hydrophobic PTFE, and hydrophilic PTFE have the sensitivity of about 2.07 pF/% RH, 1.77 pF/% RH and 0.84 pF/% RH, respectively. At high RH levels (45 to 90% RH), the high sensitivity is obtained on the sensors on the bare hydrophobic PTFE and ethanol-treated hydrophobic PTFE substrates, which is 164.26 pF/% RH and 164.98 pF/% RH, respectively, whereas the sensitivity of the sensor on the hydrophilic PTFE is only 8.02 pF/% RH. 

Since the ethanol on the ethanol-treated hydrophobic PTFE is completely evaporated during vacuum evaporation, the substrate recovers back to its hydrophobicity, and the properties of the sensor on ethanol-treated hydrophobic PTFE are very similar to that on bare hydrophobic PTFE. Compared with the hydrophilic substrate, which seriously affects the hysteresis and sensitivity of the sensor, hydrophobic PTFE is more suitable as a substrate for the humidity sensor.

### 3.7. The Result of Dynamic Characteristics

**Figure 12:**
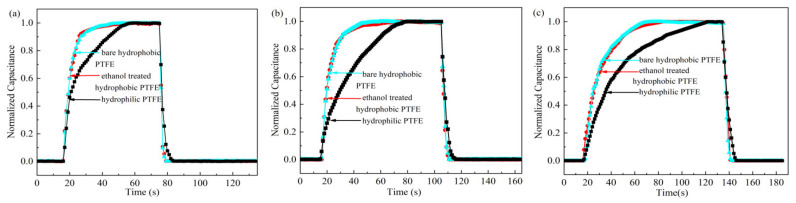
Response/recovery time of the sensors on different PTFE substrates (using N_2_): (**a**) both sides are unsealed; (**b**) the backside is sealed; (**c**) the frontside is sealed.

The dynamic responses (using N_2_) of sensors based on different PTFE substrates are shown in Figure 12a. The response/recovery time of the sensor is defined as the time to reach 90%/10% of the maximum output of the sensor as the relative humidity changes rapidly from 3% RH/96% RH to 96% RH/3% RH. After normalization of the measured capacitance value, the time when the normalized capacitance increases from 0.1 to 0.9 is defined as the response time. Similarly, the time when the normalized capacitance decreases from 0.9 to 0.1 is the recovery time.

The response/recovery time of the sensors based on bare hydrophobic PTFE and ethanol-treated hydrophobic PTFE are both 10 s/2 s. Whereas the hydrophilic PTFE substrate leads to a relatively longer response/recovery time of 29 s/5 s. The dynamic characteristic of the sensors on the hydrophobic substrates is significantly better than that on the hydrophilic substrates, which is consistent with the previous predictions.

Then, as the back side of the PTFE substrates is sealed with tape, the response/recovery time of the above sensors were measured, as shown in Figure 12b. The response/recovery time of three sensors are all extended, which are 16 s/4 s of the sensor on bare hydrophobic PTFE, 15 s/4 s of the sensor on ethanol-treated hydrophobic PTFE, and 47 s/7 s of the sensor on hydrophilic PTFE, respectively. This demonstrates that the dynamic responses of the sensors are improved by the porous PTFE substrates.

Due to the breathability of porous PTFE membranes, the sensors can detect the humidity level on the backside. As the front side is sealed, the dynamic responses of three kinds of sensors are shown in Figure 12c. The response/recovery time of sensors based on bare hydrophobic PTFE and ethanol-treated hydrophobic PTFE substrate are still similar, which are 33 s/5 s and 34 s/6 s, respectively. Whereas the hydrophilic PTFE-based sensor has longer response/recovery time of 70 s/10 s. The response speed of the sensors to the humidity through the back side is slower than that on the front side, because the water molecules need additional time to pass through the substrate and reach the sensitive layer.

Considering that the humidity sensor is used to measure humidity changes in the synthetic air in practical application, the dynamic responses of the sensors are measured using O_2_. The response/recovery time of the three kinds of sensors using N_2_ and O_2_ are listed in Table 1. Under the same experimental conditions, the response/recovery time of the same sample tested with N_2_ and O_2_ is very close. It is concluded that whether the gas is N_2_ or O_2_ has little effect on the sensor response speed.

### 3.8. Other Factors Affecting the Sensor

**Figure 13:**
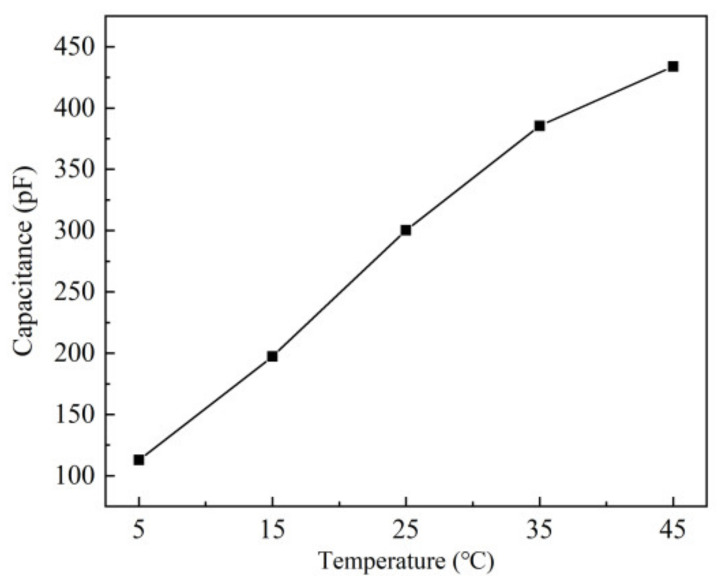
Effect of temperature on sensor capacitance under 60% RH.

The effect of temperature on the sensor based on the ethanol-treated hydrophobic substrate, which has the best performance, is studied under the condition 60% RH, the temperature varied from 5 °C to 45 °C. The results are shown in Figure 13.

As the temperature rises, the output capacitance of the sensor increases. According to the hysteresis curve of humidity sensors in Figure 10, it can be estimated that under the constant 60% RH, the capacitances of the temperature from 5 °C to 45 °C, are corresponding to 52.4% RH to 64% RH at 25 °C. Thus, the humidity offset caused by the temperature is 0.29% RH/°C.

**Figure 14:**
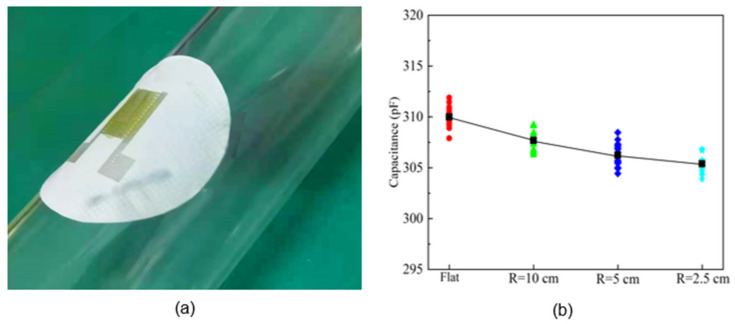
Effect of different bending conditions on sensor capacitance: (**a**) device; (**b**) results.

The effect of sensor bending is further studied. A sensor on ethanol-treated hydrophobic PTFE substrate was adhered to a flat sheet and cylinders with different radii (10 cm, 5 cm, 2.5 cm). The output capacitance of the sensor was measured at 25 °C and 60% RH, as shown in Figure 14. The average output capacitance varies about 5pF, which is small relative to the sensor sensitivity of 164.98 pF/% RH in the high humidity (45 to 90% RH) range, and thus the effect of bending is negligible. This represents an important advantage of the flexible humidity sensors: they can be placed on a bending surface without affecting their response.

### 3.9. Electrochemical Impedance Spectroscopy

**Figure 15:**
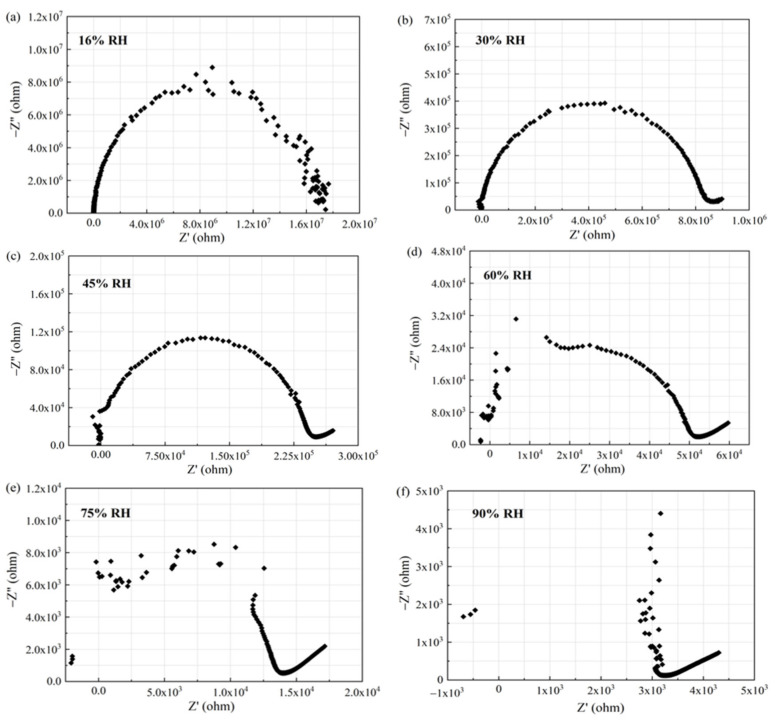
Complex impedance plots of the sensor based on ethanol-treated hydrophobic PTFE substrate under different humidity levels: (**a**) 16% RH, (**b**) 30% RH, (**c**) 45% RH, (**d**) 60%RH, (**e**) 75% RH, (**f**) 90% RH.

According to above test results, humidity sensors based on ethanol-treated hydrophobic PTFE exhibit both high yield and good sensitivity. The electrochemical impedance spectroscopy analysis is proceeded by an impedance analyzer (4294A, Agilent, Santa Clara, CA, USA). The humidity sensor is measured over a frequency range from 50 Hz to 18 MHz at different humidity levels, as shown in Figure 15. 

It can be observed from Figure 15 that the Nyquist plot of the output is a semicircle at 16% RH. At 30% RH, the radius of the semicircle is reduced, and the plot shows a tail on the right of the semicircle. As the humidity level increases (45% RH, 60% RH, 75% RH), the radius of the semicircle keeps shrinking. On the left side, the semicircle starts to be incomplete, and more points are merged into the right tail. The left semicircle does not disappear until the humidity maximum (90% RH). 

**Figure 16:**
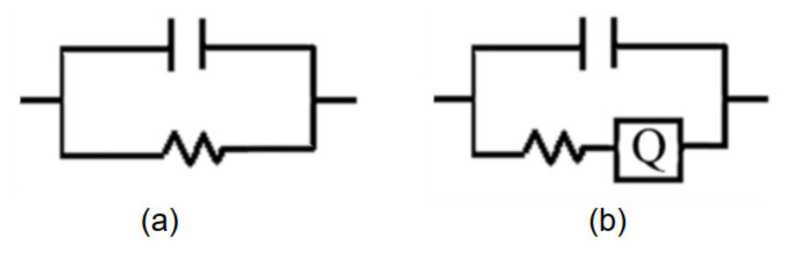
Equivalent circuits of sensor under different humidity levels; (**a**) very low RH (16% RH); (**b**) high RH (>30% RH).

The semicircle in the Nyquist diagram represents the RC parallel network, and the low frequency tail represents the Warburg impedance caused by the diffusion mechanism. The equivalent circuit diagrams are shown in Figure 16. At low humidity (16% RH), the RC parallel network is selected as the electrical model of the sensor, as shown in Figure 16a. At moderate humidity (>30% RH), a straight line of Warburg impedance is generated in the low frequency range. A constant phase angle element Q is added to improve the electrical model of the sensor, as shown in Figure 16b. 

**Figure 17:**
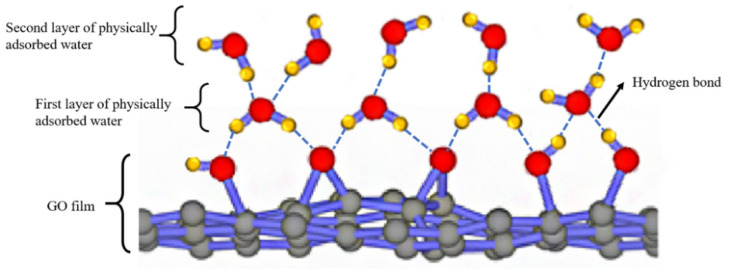
Schematic illustration for the humidity sensing mechanism of GO films. The first-layer water molecules are attached on GO through double hydrogen bonds. From the second layer, water molecules are adsorbed only through one hydrogen bond.

According to the model described in Figure 16, the Nyquist plot is fitted using the ZSimpwin software. The results are shown in Table 2. We can further explain the humidity-sensing mechanism of GO sensors on the ethanol-treated hydrophobic PTFE substrate, as shown in Figure 17. There are few free electrons inside GO. At low relatively humidity levels, water molecules are physically adsorbed on the GO surface through double hydrogen bonding on the hydroxyl group. Due to the restriction from double hydrogen bonding, water molecules cannot move freely. Additionally, the adsorbed water cannot form a continuous water layer. Although the external electric field protonates water molecules and produces the hydrogen ions (H_3_O^+^), which acts as charge carriers, the transfer of protons is difficult. The Nyquist plot is shown as a semicircle of a complete RC parallel network without Warburg impedance. It is worth noting that the plot deviates slightly from the semicircle, and exhibits the characteristic of constant phase angle. This constant phase angle behavior is primarily due to the porous structure of the electrode surface [24].

With the rising relative humidity, more water molecules are adsorbed on the GO surface and form continuous water layers, which exhibit liquid-like behavior. Through the Grotthuss chain reaction (H_3_O^+^ + H_2_O → H_2_O + H_3_O^+^) [25], protons could transport in the water layers, the diffusion based Warburg impedance occurs and has an increasing influence. As a result, the sensor shows an extremely high sensitivity [15].

## 4. Conclusions

Capacitive humidity sensors have been fabricated on porous substrates of bare hydrophobic PTFE, ethanol-treated hydrophobic PTFE, and hydrophilic PTFE. The sensors based on bare hydrophobic PTFE substrates exhibit excellent static and dynamic properties, but face the problem of low yield of the electrodes fabricated by a screen-printing process. Though the hydrophilic PTFE substrates with strong adhesion to the silver electrodes, it causes the long response time and large hysteresis of the sensors. The hydrophobic PTFE with ethanol soaking before screen-printing can improve the adhesion of the substrates to the electrodes, and maintain the same performance of the sensors as based on hydrophobic substrate. The results presented here suggest that ethanol-treated hydrophobic PTFE combines the advantages of both hydrophobic and hydrophilic PTFE substrates. 

## Figures and Tables

**Table 1:** Dynamic characteristics of the sensors using N_2_ and O_2._

Substrate State	Gas Type	Bare Hydrophobic PTFE	Ethanol Treated Hydrophobic PTFE	Hydrophilic PTFE
both sides are unsealed	N_2_	10 s/2 s	10 s/2 s	29 s/5 s
	O_2_	11 s/2 s	10 s/2 s	28 s/5 s
the backside is sealed	N_2_	16 s/4 s	15 s/4 s	47 s/7 s
	O_2_	16 s/4 s	16 s/4 s	46 s/7 s
the frontside is sealed	N_2_	33 s/5 s	34 s/6 s	70 s/10 s
	O_2_	31 s/6 s	33 s/6 s	68 s/10 s

**Table 2:** Fitting parameters in electrical models under different humidity levels.

Humidity (% RH)	R (Ω)	C (pF)	Y (S·s^−n^)	n
16	1.661 × 10^7^	2.539	-	-
30	8.354 × 10^5^	2.486	9.363 × 10^−7^	0.02595
45	5.496 × 10^4^	2.495	3.905 × 10^−6^	0.0286
60	4.885 × 10^4^	2.464	1.879 × 10^−5^	0.2561
75	1.417 × 10^4^	2.462	1.518 × 10^−5^	0.4809
90	3473	2.527	3.905 × 10^−5^	0.5335

## Data Availability

Not applicable.
